# Interplay between dietary nitrate metabolism and proton pump inhibitors: impact on nitric oxide pathways and health outcomes

**DOI:** 10.3389/fnut.2025.1648219

**Published:** 2025-09-02

**Authors:** Reem Basaqr, Abrar Babateen

**Affiliations:** ^1^Department of Clinical Nutrition, College of Applied Medical Sciences, King Saud bin Abdulaziz University for Health Sciences (KSAU-HS), Jeddah, Saudi Arabia; ^2^King Abdullah International Medical Research Center, Jeddah, Saudi Arabia; ^3^Department of Clinical Nutrition, Faculty of Applied Medical Sciences, Umm Al-Qura University, Makkah, Saudi Arabia

**Keywords:** dietary nitrate, nitric oxide, proton pump inhibitors, vascular and brain health, microbiota

## Abstract

Proton-pump inhibitors (PPIs) are often-prescribed antacids that are useful in the treatment of gastrointestinal disorders. Nonetheless, a number of studies have raised concerns about their long-term use, linking them to a higher risk of cardiovascular disease and other possible adverse effects, including brain damage. Since nitric oxide (NO) plays a vital role in neurological and vascular health, it is important to look into how PPIs might change the NO pathway. Oral bacteria and the preservation of a healthy stomach environment are essential for the external pathway’s synthesis of NO, which involves dietary nitrates (NO₃^−^) and nitrites (NO_2_^−^). PPIs have been demonstrated to decrease stomach acidity, which decreases NO bioavailability and prevents dietary NO₃^−^ from being converted to NO_2_^−^ and, subsequently, to NO. Endothelial dysfunction, which is typified by decreased vasodilation and elevated vascular resistance—two major factors in the development of hypertension—may result from this drop in NO levels. Moreover, reduced NO levels are associated with impaired brain function since NO is necessary for maintaining cerebral blood flow, neuronal transmission, and overall cognitive functioning. We propose that PPIs influence nitrate metabolism by several potential mechanisms including PPI-induced hypochlorhydria and a change in oral and gastric microbiomes leading to dysbiosis. There may also be other contributing pathways. Understanding how PPIs impact the NO₃^−^-NO_2_^−^-NO pathway is crucial for assessing their long-term effects on cardiovascular and brain health. By comprehending this connection, we may more effectively weigh the potential systemic risks of PPIs against their therapeutic advantages for gastrointestinal disorders. This may also guide safer prescription practices and patient management measures.

## 1 Introduction

Proton pump inhibitors (PPIs) are among the most widely used drugs worldwide (1), and the most potent antacid medications, as they decrease hydrochloric acid secretion in the stomach by blocking the gastric potassium-ATPase enzyme in the parietal cells. These medications are mainly recommended by physicians or gastroenterologists for gastrointestinal disorders (2), particularly for gastroesophageal reflux disease (3). The other diseases PPIs are prescribed for are heartburn, esophagitis, *Helicobacter pylori* infections, peptic ulcers, and Zollinger-Ellison syndrome (3). There are several PPIs available, including omeprazole (Losec®), lansoprazole (Prevacid®), pantoprazole (Protonix®), rabeprazole (Aciphex®), and esomeprazole (Nexium®)—the S-enantiomer of omeprazole (4).

Reports of possible side effects have increased since PPIs were first put on the market, because many people start taking them on their own and continue to take them for longer than is advised, sometimes without a doctor’s supervision. According to the US Food and Drug Administration (FDA), fractures of the hip, wrist, and spine, hypomagnesaemia, kidney problems, and cardiovascular illnesses are among the possible dangers associated with PPI use (5, 6). Therefore, stricter adherence to clinical guidelines is essential to minimize overuse and enhance patient safety. The American College of Gastroenterology (ACG) determined that the duration of PPI use for esophageal reflux disease should not exceed 8 weeks.

Dietary nitrate (NO₃^−^), predominantly found in plant-based foods such as leafy greens and root vegetables, plays a crucial role in maintaining cardiovascular health. Its primary mechanism of action is mediated via its conversion to nitric oxide (NO), a potent vasodilator that supports vascular function and regulates blood pressure. NO helps to relax and widen blood vessels, which enhances blood flow, reduces arterial stiffness, and improves endothelial function—all of which are essential factors for maintaining optimal blood circulation and preventing cardiovascular and cerebrovascular diseases (6, 7).

Recent concerns have arisen regarding the potential of long-term use of PPIs to disrupt the pathway through which NO₃^−^ is converted to nitrite (NO_2_^−^) then to NO (known as the NO₃^−^– NO_2_^−^–NO pathway) (8). This disruption may hinder the beneficial cardiovascular and cerebrovascular effects associated with dietary NO₃^−^ consumption (9, 10). To our knowledge, only one clinical trial has explicitly looked at how PPIs affect the cardiovascular benefits of NO₃^−^ (8), even though the long-term safety of PPI use has recently attracted a lot of scientific attention due to large cohort studies that linked PPI use to negative health outcomes, such as an increased risk of cardiovascular and cerebrovascular disease (8).

Montenegro et al. carried out a randomized, double blind, placebo-controlled study with 15 healthy volunteers and showed that pretreatment with esomeprazole considerably reduced the hypotensive effects of sodium NO_2_^−^ (8). Although sodium NO_2_^−^ reduced systolic blood pressure by an average of 6 ± 1.3 mm Hg after a placebo, this effect was significantly reduced in patients who received esomeprazole (8). Collectively, these studies demonstrate how important stomach acidity is in modulating the effects of dietary NO₃^−^ on the cardiovascular system.

This review hypothesizes that impaired NO bioavailability from the NO₃^−^– NO_2_^−^–NO pathway may contribute to an increased risk of hypertension and other negative consequences, such as cognitive decline. PPIs may reduce the benefits of dietary NO₃^−^ by altering stomach acidity, which lowers NO bioavailability.

## 2 Brief description of the search strategy

This narrative review aims to elucidate the mechanistic interactions between PPI use and the NO₃^−^–NO₂^−^–NO pathway, with particular attention to implications for cardiovascular and cerebrovascular health. We chose the narrative review format because of its flexibility in synthesizing diverse types of evidence, including mechanistic, microbial, pharmacological, and clinical studies. Both authors performed the primary search in the literature using the electronic databases PubMed and Google Scholar for publications in the English language published any time up to July 2025. Combinations of the following keywords were used in the search: “proton pump inhibitors,” “dietary nitrate,” “nitrite,” “nitric oxide,” “oral microbiota,” “gastric juice,” ““cardiovascular health,” “blood pressure,” and “cognition” or “cognitive function.” Additional articles were identified through manual screening of reference lists in relevant publications.

Studies mentioned in Table 1 were selected based on their relevance to the mechanistic focus of the review. Emphasis was placed on recent primary research articles, high-quality reviews, and publications offering insight into the cardiovascular, or neurological effects of PPIs or nitrate metabolism. No formal inclusion or exclusion criteria were applied. This approach ensured a broad, integrative synthesis of the current evidence base while highlighting key knowledge gaps and future research directions.

**Table 1:** Summary of studies on the impact of PPIs and acid-reducing medications on the NO₃–NO pathway and blood pressure regulation.

Reference	Study design	Objective	Intervention	Outcome measured	Findings
Pinheiro et al. (114)	Experimental study (Wistar rats)	Evaluate the impact of omeprazole pretreatment on the hypotensive effects of NaNO_2_^−^ in animals	A single dose of vehicle (1 mL/kg 2% Tween 80) or omeprazole (30 mg/kg) + NaNO_2_^−^ (15 or 45 mg/kg) by gavage.	1/Plasma NO*_x_* (NO₃^−^ + NO_2_^−^) 2/Gastric pH 3/Vascular reactivity	1/Omeprazole pretreatment reduced the hypotensive effects of oral, but not intravenous, sodium nitrite, suggesting that oral NaNO_2_^−^ effects depend on NO formation in the stomach. 2/The attenuation with omeprazole is not due to differences in plasma NO_2_^−^, NOx, or S-nitrosothiol levels.
Amaral et al. (102)	Experimental study (induced hypertension, Wistar rats)	Examine the hypotensive effects of NaNO_2_^−^ in induced hypertension rats that were treated with TEMPOL or vehicle Secondary objective: To investigate the impact of gastric pH on the hypotensive effects of NO_2_ induced by TEMPOL.	1/TEMPOL (18 mg/kg; po) + cumulative doses of NaNO_2_^−^ (0, 0.16, 0.5, 1.6, 5, and 15 mg/kg) or vehicle 2/Omeprazole (30 mg/kg; po)	1/MAP 2/ Plasma NO*_x_* (NO₃^−^ + NO_2_^−^) 3/ Gastric pH	Pretreatment with omeprazole reduces the hypotension caused by NaNO_2_^−^ and diminishes the effects of TEMPOL.
Pinheiro et al. (103)	Experimental study (2K1C) hypertensive rats	To assess the hypothesis that the antihypertensive effects of orally administered NO_2_^−^ or NO₃^−^ are mediated by the production of S-nitrosothiols and to explore how these effects are affected by changes in gastric pH	NaNO_2_^−^ (15 mg/kg) or NaNO_3_^−^ (140 mg/kg) +omeprazole (10 mg/kg i.p. or vehicle) daily, by gavage.	1/SBP 2/ MAP 3/ Gastric pH 4/ Plasma NO₃^−^, NO_2_^−^, and S-nitrosothiols	Chronic omeprazole use abolishes NaNO_2_^−^ antihypertensive effects without altering plasma NO_2_^−^ or NO₃^−^, highlighting gastric acidity’s role in their efficacy
Montenegro et al. (8)	RCT, DB, CR study (15 healthy nonsmoking, normotensive subjects, aged 19–39 years)	To evaluate the acute effects of orally ingested NO_2_^−^ on blood pressure in healthy volunteers with or without esomeprazole pretreatment to elevate gastric pH.	Placebo or esomeprazole (40 mg) 16, 8, and 1 h before intake of NaNO_2_^−^ (0.3 mg/kg)	1/BP 2/ Plasma NO₃^−^, NO_2_^−^	NO_2_^−^ reduced SBP by 6 ± 1.3 mm Hg after placebo, but this effect was diminished with esomeprazole pretreatment.
Christopher Eff (115)*	RCT, DB, CR study (6 healthy subjects)	To evaluate the effect of PPI on NO₃^−^ bioactivation during submaximal exercise	NaNO_3_^−^(10 mg/kg). Placebo or esomeprazole (10 mg) were taken 24 h, 12 h and directly before being tested.	1/VO₂, VCO₂ 2/RER 3/VE 4/Lactate 5/Glucose, 6/HR 7/BP 8/Venous NO₃^−^	No significant differences between treatments.
Sanches-Lopes et al. (10)	Experimental study (hypertensive rats)	To evaluate the blood pressure responses to oral NO_2_^−^ in hypertensive rats administered omeprazole and ranitidine	Vehicle (1 mL/kg 2% Tween 80), or with the ranitidine (100 mg/kg), or with omeprazole (30 mg/kg) + NaNO_2_^−^ (15 mg/kg; orally)	1/MAP 2/Gastric pH 3/Gastric NO	Both drugs raised gastric pH, impaired oral NO_2_^−^-induced hypotension and gastric NO production, and reduced RXNO levels without affecting plasma NO_2_^−^ or NO₃^−^

*Master’s thesis. BP, blood pressure; DB, double blind; CR, crossover; HR, heart rate; NO₃^−^, nitrate; NO_2_^−^, nitrite; MAP, mean arterial pressure; RER, respiratory exchange ratio; RCT, randomized controlled trial; SBP, systolic blood pressure; 2K1C, two-kidney, one-clip; VO₂, volume of oxygen consumption; VCO₂, volume of carbon dioxide production, VE, minute ventilation.

## 3 Overview of NO pathways with focus on the NO_3_^−^–NO_2_^−^–NO pathway

**Figure 1:**
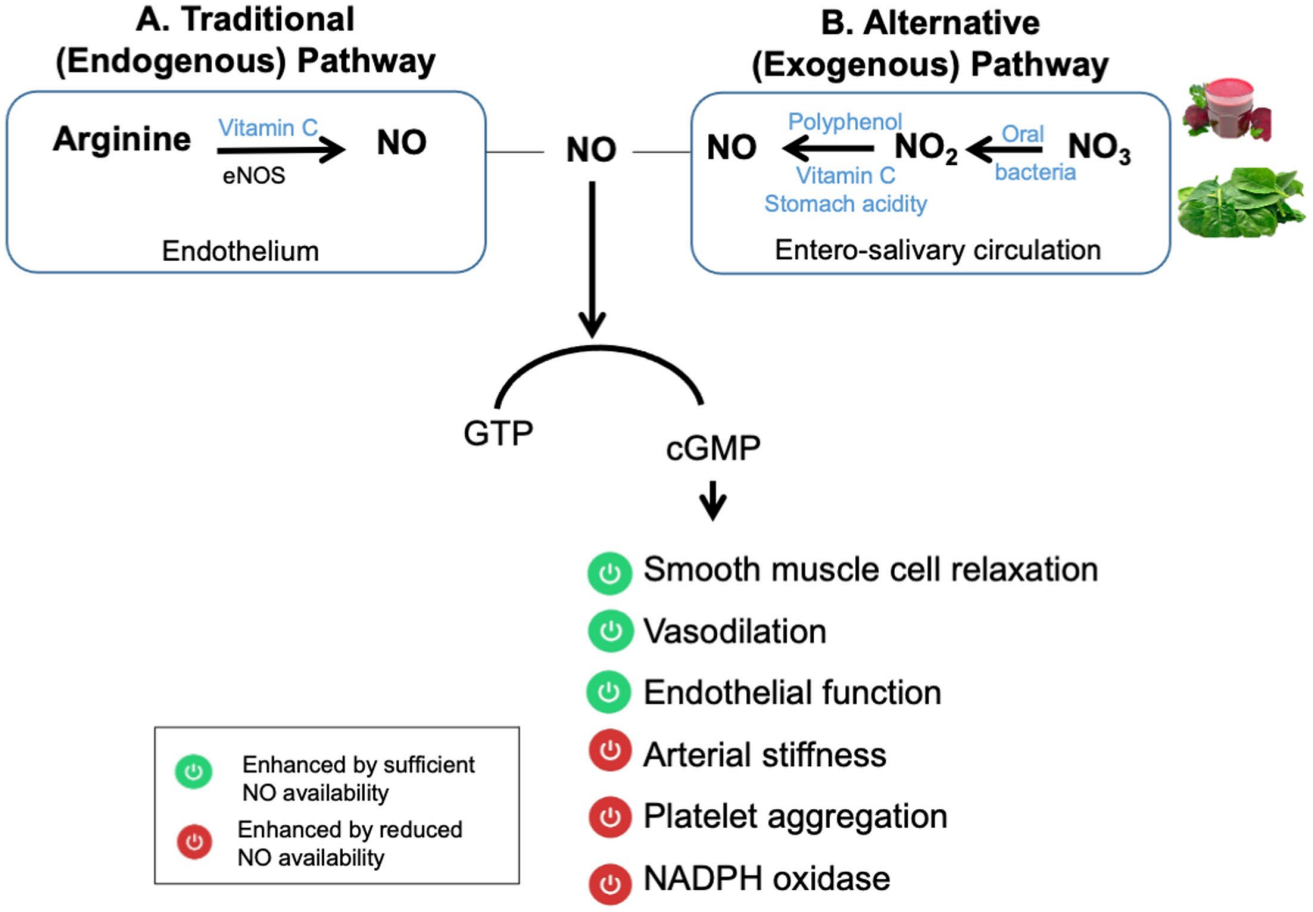
Overview of the two major pathways producing nitric oxide. In the traditional pathway, NO is synthesized from L-arginine via endothelial nitric oxide synthase (eNOS). The alternative pathway involves dietary nitrate (NO₃^−^), which is reduced to nitrite (NO_2_^−^) by oral bacteria, then to NO under acidic or hypoxic conditions. This pathway bypasses NOS and is especially important when the classical pathway is impaired. Once formed, NO activates soluble guanylate cyclase, which converts guanosine triphosphate (GTP) into cyclic guanosine monophosphate (cGMP), a key second messenger mediating many downstream effects. The green-marked functions in the figure are enhanced by adequate NO bioavailability. The red-marked functions represent physiological impairments associated with reduced NO levels.

**Figure 2:**
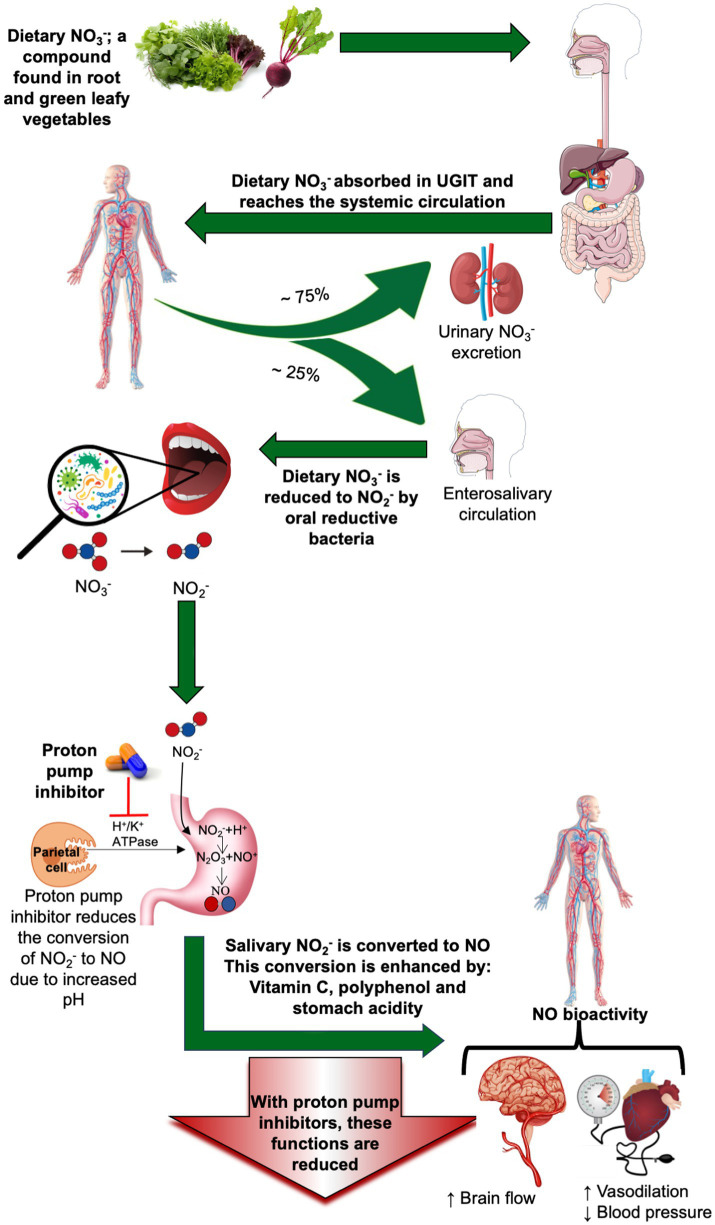
Overview of the nitrate-nitrite-nitric oxide pathway and the inhibitory role of proton pump inhibitors and its associated health functions. The exogenous pathway is enzyme-independent and involves the enterosalivary conversion of dietary nitrate (NO_3_^−^) to nitrite (NO_2_^−^) and then to nitric oxide (NO). It involves two sequential reductions. The first step is the reduction of NO₃^−^ to NO_2_^−^ following dietary consumption of NO₃^−^-rich foods like beetroot and green leafy vegetables. Dietary NO₃^−^ is rapidly absorbed within 30 min, reaching peak plasma levels approximately 1-h post consumption. Dietary NO₃^−^ is rapidly absorbed in the upper gastrointestinal tract. About 75% of the absorbed NO₃^−^ is excreted in urine, while approximately 25% is concentrated in saliva. Facultative anaerobic bacteria in the oral cavity, which possess NO₃^−^ reductase enzymes, reduce NO₃^−^ to NO_2_^−^ under hypoxic conditions over a few hours. The second step in the exogenous pathway is the reduction of NO_2_^−^ to NO and other nitrogen oxides upon systemic absorption. When NO_2_^−^ is swallowed and enters the acidic gastric environment, a small proportion is reduced to NO, while the majority of the salivary NO_2_^−^ is absorbed into the circulation, where it can be further reduced to NO by specialized enzymes with reductase activity such as xanthine oxidoreductase, carbonic anhydrase, and aldehyde oxidase. These reactions are greatly enhanced during conditions like hypoxia and low pH levels and in the presence of polyphenol and vitamin C. This process supports systemic NO bioavailability, benefiting vascular and cognitive health. However, proton pump inhibitors (PPIs) suppress gastric acid secretion, impairing NO_2_^−^ to NO conversion and reducing NO-mediated physiological functions. N_2_O_3_: dinitrogen trioxide.

NO is a crucial signaling molecule that is involved in many physiological processes. Sufficient NO production is fundamental for the maintenance of a healthy vascular system. It is produced through two main pathways: the endogenous and exogenous pathways (11), summarized in Figure 1. The endogenous pathway, an enzyme-dependent process involving endothelial NO synthase (eNOS), synthesizes NO from the amino acid L-arginine. However, under local hypoxic conditions, the efficiency of this pathway is compromised (12). As a result, increasing attention has shifted toward the exogenous, enzyme-independent, pathway in recent years, which serves as an alternative pathway for NO production. This pathway is largely influenced by diet and microbial activity. Specifically, commensal bacteria such as Neisseria, Veillonella, Kingella, Pseudopropionibacterium and Propionibacterium, Streptococcus, Prevotella, and others are in the oral cavity play a critical role in the reduction of dietary NO₃^−^ to NO₂^−^ and, subsequently, to NO, especially under hypoxic conditions via two sequential reduction steps (13) (see Figure 2). These bacteria increase in number with nitrate consumption according to clinical studies (14–16).

After ingestion of NO₃^−^-rich foods, such as beetroot and leafy greens, NO₃^−^ is rapidly absorbed and peaks in plasma within around 1 h. In the bloodstream, NO₃^−^ combines with the body’s naturally produced NO₃^−^, primarily originating from the L-arginine-NO pathway. Roughly 75% is excreted in urine, while about 25% is actively secreted in the saliva after being enriched in the salivary glands and then recirculated by the enterosalivary circulation. In the oral cavity, oral facultative anaerobic bacteria reduce about 20% of salivary NO₃^−^ to NO_2_^−^ under hypoxic conditions (14). The second step occurs in the acidic environment of the stomach, where most of the NO_2_^−^ (pKa 3.4) is protonated to nitrous acid (HNO_2_); the latter decomposes into NO and other nitrogen oxides upon systemic absorption (17). NO generation in the stomach is promoted by antioxidants such as vitamin C and polyphenol that facilitate reactions between NO₂^−^ and these reducing agents, leading to muscle relaxation (18, 19). However, data on the relative contribution of these agents to NO production remain limited (20). In systemic circulation, several enzymatic pathways enhance the conversion of the remaining NO_2_^−^ to NO, particularly under acidic and hypoxic conditions (18). Enzymes such as xanthine oxidoreductase, carbonic anhydrase, and aldehyde oxidase, along with polyphenols and vitamin C, play key roles (21).

There is emerging research highlighting the role the human microbiota plays in the production of NO directly or indirectly. A recent study suggests that specific gut microbes, such as *Akkermansia muciniphila*, can enhance systemic NO production (22); in an in-vitro experiment, *A. muciniphila* was shown to increase nitrite levels, a marker of NO production, in macrophages, indicating its potential role in modulating NO-related immune responses (22). Furthermore, gut microbial communities are capable of synthesizing NO from dietary nitrate using the dissimilatory nitrate reduction to ammonium (DNRA) pathway (23). This is a bacterial alternative to the body’s typical NO synthesis pathways. *In vitro* studies on human gut microbiota from fecal samples have revealed that exposure to NO can reshape microbial diversity and metabolism, reinforcing the two-way relationship between NO and microbiota composition (24).

Overall, the two main factors in the exogenous pathway are oral bacteria and the acidic gastric environment. This pathway serves as a backup system for NO generation, especially when the classic, endothelial NO-synthase-dependent, pathway is compromised due to NO scavenging by reactive oxygen species in the presence of cardiovascular risk factors (18). Therefore, NO₃^−^ from the diet and oral microbes offers an early defense against NO deficiency (13).

Alterations in oral microbiota composition have been believed to influence the overall production of nitric oxide (13). Recent studies have highlighted that mouthwash or the overuse of antibiotics can impair the role of oral bacteria involved in the first step of dietary NO production (21), while PPIs impair the second step by blocking NO_2_^−^ reduction—both of which can potentially lead to NO deficiency (25). Therefore, stomach health is essential for maximizing the benefits of dietary NO₃^−^.

## 4 Role of the NO₃^−^–NO_2_^−^–NO pathway in biological processes

The NO₃^−^- NO_2_^−^-NO pathway supports gastric, cardiovascular, and cognitive functions (26). By producing NO, it enhances blood flow and oxygen delivery and reduces oxidative stress, inflammation, and immune dysfunction (27). This pathway offers a potential point for therapeutic strategies, especially when the NOS-dependent system is compromised (18). Alongside these functions, NO also has a role in esophageal motility. Dysregulation of NO signaling may contribute to neurotoxic effects that impact esophageal function (28), indicating a potential area for further research. The following sections discuss how NO₃^−^ and NO_2_^−^ contribute to stomach, cardiovascular, and brain health.

### 4.1 Stomach health

Dietary NO₃^−^ serves a protective function in the digestive system, by boosting the antimicrobial properties of the stomach (29). As mentioned previously, in the acidic conditions of the stomach, NO_2_^−^ transforms into nitrous acid (HNO_2_), which subsequently breaks down to NO and various nitrosating agents, such as dinitrogen trioxide (N_2_O_3_) and nitrosonium ion (NO^+^) (30). These agents promote the formation of S-nitrosothiols, which play a protective role in stomach health. S-nitrosothiols not only act as stable NO donors, sustaining NO bioactivity, but also mediate S-nitrosylation, a key post-translational modification that involves the covalent addition of NO groups to protein cysteine residues (30). NO signaling molecules released by resident gut microbiota can induce extensive S-nitrosylation modifications to the host proteome, including argonaute proteins, which regulate host gene expression and development via microRNAs (31). This highlights the complex regulatory roles that nitrogen signaling pathways play in maintaining gastrointestinal health.

NO plays a critical role in killing or inhibiting harmful microbes such as *Escherichia coli* and *Candida albicans*, thereby strengthening the stomach’s defense against infections (32). Moreover, it is known that *H. pylori* can survive in highly acidic conditions, but it is inhibited when NO_2_^−^ levels reach a threshold of about 1 Mm (33). This suggests that consuming NO₃^−^-rich foods may help prevent *H. pylori* colonization, although no epidemiological studies have confirmed this link (33). Dietary NO₃^−^ also enhances gastric mucosal blood flow and increases mucus thickness, improving nutrient and oxygen delivery to the stomach lining and supporting tissue repair (34). This is consistent with research showing that after microbial dysbiosis, levels of the tight junction proteins claudin and occludin fall, but that these levels can be recovered by increasing dietary NO₃^−^ intake (35). Additionally, dietary NO₃^−^ protects against gastric damage and ulceration caused by nonsteroidal anti-inflammatory drugs and stress (36, 37). In animal models, NO₃^−^ supplementation increased mucus thickness and upregulated *MUC6* expression, a vital component of the gastric mucosal barrier (38). Protective mechanisms may also include the reduction of inflammation in the gastrointestinal tract (39). Although the processes by which NO₃^−^ metabolism contributes to the resolution of gut inflammation are not fully understood, they may relate to equilibrium between the generation of reactive nitrogen species and the maintenance of intestinal integrity (39).

### 4.2 Cardiovascular health

Most of the evidence linking dietary NO₃^−^ and NO_2_^−^ to health benefits focuses on cardiovascular disease and its risk factors, particularly hypertension. Reduced NO bioavailability and lower plasma NO_2_^−^ levels are markers of impaired NO homeostasis, which is commonly observed in conditions such as aging, hypercholesterolemia, hypertension, and other cardiovascular-related diseases (40). Most clinical studies published on inorganic NO₃^−^ support the notion that NO₃^−^ supplements NO production exogenously to reverse NO homoeostasis, and focus on its effects on reducing blood pressure (17, 41–43), reversing vascular dysfunction in older adults (44), and improving angiogenesis following chronic ischemia through the restoration of NO homeostasis (11, 20, 45, 46). Most human studies have used standardized beetroot juice to determine NO₃^−^ levels and found that 150 to 550 mg of NO₃^−^ needs to be administered 90 min before measurable endpoints to allow for enterosalivary circulation and the conversion of NO_2_^−^ and NO to present improvements (26).

In the last 10 years, numerous systematic reviews and meta-analyses have indicated the clinical effectiveness of NO₃^−^ supplementation in significantly lowering blood pressure, especially systolic blood pressure (17, 41–43, 47–49). However, most trials involved healthy participants, limiting the evidence for those with cardiovascular risk factors like hypertension or high cholesterol (41). In addition, research has highlighted various pathways through which dietary NO₃^−^ supports cardiovascular health including enhancing endothelial function (50, 51), regulating sympathetic nervous system activity (49), suppressing NADPH oxidase activity (52), modulating angiotensin II receptor signaling (53), reducing arterial stiffness (44), inhibiting platelet aggregation (54), activating soluble guanylate cyclase, and promoting the release of cyclic GMP (55). Furthermore, the intake of dietary NO₃^−^ has been linked to the stabilization of atherosclerotic plaques (56). Thus, dietary NO₃^−^ lowers the risk of atherosclerosis via multiple synergistic mechanisms, ultimately contributing to cardiovascular health.

### 4.3 Cognitive health

Vascular health and cerebrovascular blood flow play a significant role in maintaining cognitive function (57), as emphasized by the American Heart Association (AHA) and American Stroke Association (ASA) (58). Vascular risk factors are strongly associated with an increased likelihood of developing dementia, including Alzheimer disease and vascular dementia (59). NO supports brain homeostasis, as it significantly influences cerebrovascular blood flow, which helps support cognitive function (60, 61).

Previous studies have shown that dietary NO₃^−^ supplementation has beneficial effects on cognitive abilities and motor skills via the enhancement of NO production (62). These benefits are proposed to be linked to increased cerebral blood flow and improved cellular metabolism efficiency (62, 63). However, in 2018 a meta-analysis found insufficient evidence due to study limitations such as small sample sizes, short duration, and the use of predominantly healthy participants, which may have affected the outcomes (64, 65). Although some findings are encouraging, the evidence remains inconsistent (65).

There is currently no correlation between cognitive function and NO₃^−^ levels, whether determined by urinary NO₃^−^ concentrations or dietary intake (66, 67). Interestingly, a recent 10-year cohort study of 1,254 older adults reported improved cognitive performance with plant-based NO₃^−^ intake, although it relied on baseline data only, highlighting the need for further research into underlying mechanisms (68).

## 5 Adverse health outcomes of PPIs associated with vascular function

PPIs are frequently used by patients with cardiovascular disease to prevent gastrointestinal bleeding, a major side effect of antithrombotic drug therapy. Extensive PPI use has been linked to serious adverse events including anemia, fractures, renal damage, and increased infection risk (69, 70). Given that the influence of PPIs on cardiovascular and cerebrovascular health is the focus of this review, some of the adverse effects that contribute to these conditions are discussed.

### 5.1 PPIs and endothelial dysfunction

The endothelium, a monolayer of cells lining blood vessels, forms an interface between the vessel wall and the lumen (71). It plays a crucial role in regulating cardiovascular homeostasis, by releasing vasodilators such as NO, prostacyclin I2 (PGI2), and different endothelium-derived hyperpolarizing factors (72). NO is the master signaling molecule that plays a crucial role in regulating vascular tone and permeability, the proliferation of smooth muscle cells, and blood fluidity. Overall, vascular health depends on the integrity of endothelial function (48, 73).

Endothelial dysfunction (ED) is reversible, and it is the first key stage of atherosclerosis. Numerous factors, such as physical trauma or stress from direct trauma, turbulent blood flow, or toxins (reactive oxygen species) from the presence of cardiovascular risk factors, such as obesity, hypertension, hyperlipidemia, or smoking can impair endothelial function (74). These factors impair endothelial function by promoting the release of vasoconstrictors, proinflammatory, and procoagulant chemicals, leading to platelet aggregation (75, 76). Overall, ED is largely a result of reduced NO bioavailability, due to either reduced synthesis or increased degradation, which increases intracellular calcium levels within smooth muscle cells, leading to contraction (77).

Analogs of guanidino-substituted L-arginine bind to eNOS and competitively limit NO production. A natural NO synthase antagonist called asymmetric dimethylarginine (ADMA), found in plasma and urine, is important in ED as it reduces vasodilation, increases platelet aggregation, and improves monocyte adhesion (78). ADMA is generated during protein metabolism and degraded by dimethylarginine dimethylaminohydrolase (DDAH), the dysfunction of which is the main cause of elevated ADMA levels (79). Studies suggest that prolonged PPI use may inhibit DDAH activity, decreasing NO production and impairing endothelial function, as seen with omeprazole and lansoprazole (80, 81). Furthermore, in-vitro studies revealed that whereas esomeprazole and pantoprazole do not exhibit this pattern, lansoprazole, omeprazole, and rabeprazole raise ADMA levels through DDAH inhibition (82). Long-term PPI use is linked to decreased NO availability and ED in healthy people according to a cross-sectional study (81). Additionally, elevated ADMA levels have been noted in hypertensive patients, indicating a connection between hypertension and ED (78, 83). Esomeprazole has been shown to reduce the activity of endothelium lysosomal enzymes and telomere-preserving genes, while increasing markers of cellular aging (84). These observations link long-term PPI use to an elevated risk for cardiovascular disease and hypertension, highlighting the possible role of PPIs in vascular dysregulation.

### 5.2 PPIs and vitamin B deficiency

Vitamin B12 absorption takes place on parietal cells in the gastric mucosa, which secrete intrinsic factors for B12 uptake. Lam et al., reported that prolonged use of antacids may increase the risk of developing vitamin B12 deficiency (85). Vitamin B12 is essential for converting homocysteine into methionine, and its deficiency results in hyperhomocysteinemia, which is associated with vascular impairment and hypertension (86).

Increased homocysteine levels negatively influence cardiovascular function by impairing vasodilation, enhancing oxidative stress, promoting the proliferation of smooth muscle cells, and compromising blood vessel flexibility, ultimately contributing to the development of hypertension. Homocysteine also reduces NO bioavailability by interfering with ADMA and increasing the production of reactive oxygen species, further compromising vascular function (87–89).

### 5.3 PPIs and hypocalcemia

Calcium is primarily absorbed in the small intestine. The acidic environment of the stomach is essential for its ionization for best absorption (90). PPI use may raise the pH of the stomach, which could decrease calcium ionization, hindering its absorption and raising the risk of deficiencies (91). In line with decreased intestinal calcium absorption brought on by changed gastric pH, a recent comparative cross-sectional investigation found that 40 patients taking omeprazole had significantly lower plasma calcium levels in comparison with 50 healthy controls (92). This could raise the risk for osteoporosis, fractures, and bone demineralization, underscoring the necessity to monitor calcium status in long-term PPI users (93). Furthermore, calcium deficiency stimulates parathyroid hormone secretion, which is associated with arterial stiffness and impaired vasodilation and, thus, may promote elevated blood pressure (93).

### 5.4 PPI use and hypomagnesaemia

Magnesium is a vital mineral that plays a crucial role in various physiological functions. PPIs are also believed to hinder the intestinal absorption of magnesium, potentially leading to long-term magnesium deficiency and disruption of the body’s magnesium homeostasis (94). A recent cross-sectional study of hospitalized patients using PPIs for at least 6 months found that 36% had hypomagnesemia (≤1.7 mg/dL) at admission (95). PPI-related hypomagnesemia was associated with chronic kidney disease, impaired tubular function, anemia, hyponatremia, malignant bone compromise, vasoconstriction, and endothelial dysfunction, highlighting the potential risks of long-term PPI use on magnesium homeostasis (95). These findings emphasize the importance of monitoring magnesium levels in long-term PPI users, as persistent deficiency may contribute to increased cardiovascular risk.

### 5.5 PPI use and cognitive decline

As the use of PPIs is increasing, concerns about their safety, particularly regarding cognitive function, have arisen. According to a recent systematic review of 11 studies, most of the studies relating PPI use to cognitive decline discovered a strong correlation with acute cognitive impairment (96). To fully comprehend the connection between PPI use and cognitive function, particularly in older persons, additional longitudinal research is necessary (96). In addition, a recent large cohort study revealed that PPI users were more likely than nonusers to have dementia from all causes (97).

## 6 Interaction between PPIs and the NO₃^−^–NO_2_^−^–NO pathway

Although PPIs can effectively manage acid-related gastrointestinal disorders, they may lead to unintended consequences on NO metabolism and gastric physiology. PPIs are widely prescribed to reduce gastric acidity by inhibiting the hydrogen-potassium ATPase enzyme in gastric parietal cells (98). This suppression relieves acid-related conditions and promotes mucosal healing, promoting recovery from inflammation and erosive damage (99). However, by raising gastric pH, PPIs may impair dietary NO₃^−^ and NO_2_^−^ conversion to NO, which requires an acidic environment (100). Studies show that PPIs significantly reduce gastric NO production, flow-mediated dilation, and plasma citrulline, markers of NO availability, despite NO₃^−^ intake (81). This could weaken NO-mediated cardiovascular benefits. Lundberg et al. found that gastric NO production, measured using ozone chemiluminescence, was significantly reduced by about 95% after high-dose omeprazole administration, regardless of NO₃^−^ intake from lettuce (8). In addition, preclinical and cohort studies further confirm that acid suppression blunts NO-related blood pressure effects and reduces bioactive nitrogen compounds. For instance, in a recent cohort study of 1,298 participants, PPI users had significantly lower flow-mediated dilation and plasma citrulline levels, indicating reduced NO production (81). Similarly, a more recent prospective cohort study among 64,720 postmenopausal women reported an increased risk of hypertension associated with regular PPI use. This adverse outcome on vascular function may be influenced by the long-term suppression of gastric acid production, possibly by disruption of the NO pathway (101).

Furthermore, recent research sheds light on the many ways acid-suppressing medications affect NO bioavailability following NO₃^−^ administration. In a recent preclinical study, researchers assessed the effects of omeprazole and ranitidine in rats, showing how changes in stomach pH influence NO production and bioavailability following the administration of NO₃^−^, providing strong evidence that acid-reducing medications effectively abolish the blood-pressure-lowering effects of oral NO_2_^−^ treatment (10). This aligns with studies that demonstrate how PPIs, by altering gastric pH, disrupt oral NO₃^−^–induced hypotensive responses and reduce gastric NO production (summarized in Table 1).

Drawing on insights from mouthwash studies, where differences in strength and composition led to varying impacts on oral bacteria, NO₃^−^ reduction, and the hypotensive effect of dietary NO₃^−^ (102, 103), it is reasonable to hypothesize that the strength of acid suppression by different drugs may result in varying degrees of disruption of NO₃^−^ conversion. Compared with H_2_ blockers, PPIs have a stronger impact, with clinical implications for patients relying on dietary NO₃^−^ for vascular health. Recent research, however, shows that ranitidine and omeprazole both decrease stomach NO production and hinder oral NO_2_^−^–induced hypotensive responses (10). Additionally, these medications reduce the rise in reactive nitrogen oxide compounds, such as S-nitrosothiols (10), which are critical for NO bioactivity. It is interesting to note that neither seems to have a major impact on blood concentrations of NO₃^−^. This implies that the local gastric conversion processes and the ensuing acute cardiovascular effects are significantly hampered by the increase in gastric pH, even though the systemic availability of these chemicals may stay largely unchanged.

Given the heavy reliance of the exogenous pathway on the oral microbiota and its association with various cardiometabolic benefits when stimulated by dietary NO₃^−^, it is essential to consider factors that affect gut microbial balance. Both dietary habits and medications, notably PPIs, can disrupt this balance (104). There have been recent reports that hypochlorhydria in PPI users is raising their risk of infections (105), which could be attributed to dysbiosis, a disruption of the composition and diversity of microbes in the intestines and oral cavity brought on by long-term PPI use (105–107). Normally, the stomach’s acidity serves an essential role in removing harmful bacteria and protecting against enteric infections (108). PPI use has been associated with a reduction in the diversity of the microbiota and an increase in potentially harmful microbes such as *Enterococcus*, *Streptococcus*, and *Clostridium difficile*, while beneficial species that reduce NO₃^−^ may be supressed (105, 109, 110). Xiao et al. observed significant alteration in the composition of gut microbiota with short-term PPI use, mainly by translocation of bacteria originally found in the oral cavity, such as *Streptococcus anginosus*, to the gut. This shift was mitigated by chlorhexidine mouthwash, suggesting that this oral-to-gut microbial shift may be how PPIs disrupt microbial NO₃^−^ metabolism (106). There have been similar reports of microbiota shifts related to PPI use (105). Notably, strong suppression of acid secretion in the stomach may increase potentially carcinogenic N-nitroso compounds due to an increase in the number of bacteria, as indicated in earlier studies (111). Collectively, these findings provide evidence that dysbiosis induced by PPI use may impair NO bioavailability through the reduction of gastric acidity as well as disruption of the gut microbial ecosystem responsible for NO₃^−^ metabolism, thereby affecting microbial NO₃^−^ reduction capacity.

In the oral cavity, inorganic NO₃^−^ can boost NO₃^−^-reductase activity by increasing salivary flow and suppressing acid-producing bacteria; however, as earlier mentioned, PPI use may suppress beneficial NO₃^−^-reducing species (109, 110). This state of dysbiosis is characterized by a reduction in NO₃^−^-reducing bacteria and reduced NO_2_^−^ production and NO bioavailability, which has been linked to cardiometabolic disorders (109). These findings highlight how the microbiota contributes to host homeostasis and emphasizes the importance of cautious long-term use of PPIs. It is recommended to follow strategies to maintain or restore the oral and gut microbiomes to support the integrity of the NO₃^−^–NO₂^−^–NO pathway and decrease the risk of cardiometabolic complications (15, 112).

Disruption of NO₃^−^ metabolism by PPIs may occur by several interconnected pathways. Firstly, hypochlorhydria induced by PPIs raises gastric pH, diminishing NO production by diminishing the conversion of NO₃^−^ to NO₂^−^ in the stomach (10, 105, 113). Secondly, PPIs contribute to alteration of the gut microbiota by facilitating the translocation of NO₃^−^-reducing bacteria (such as *Streptococcus* and *Enterococcus*) to the gut due to decreased gastric acid secretion (110). Additionally, the resulting dysbiosis may lead to suppression of beneficial NO₃^−^-reducing species and potentially shift nitrogen toward alternative metabolic pathways like ammonia production (105, 110). Combined, these alterations may lower NO bioavailability by reducing entero-salivary NO₃^−^ - NO₂^−^ recycling.

## 7 Future directions

The effects of PPI use on dietary NO₃^−^ metabolism as well as vascular and cognitive health are still being investigated in this new field of study. Observational studies are crucial to determine how long-term PPI use may disrupt the conversion of dietary NO₃^−^ to NO and the ensuing consequences on NO bioavailability, even though they are unable to verify causal relationships. An effective first step in examining the connection between PPI use and NO₃^−^ metabolism is to leverage current cohort studies with detailed dietary, medication, and biomarker data (e.g., plasma/urinary NO₃^−^, NO_2_^−^, and NO levels) to assess the impact of chronic PPI use on NO bioavailability. This strategy may have an impact on cognitive performance and vascular health, particularly in people who are more susceptible to neurodegenerative or cardiovascular problems. There is an urgent need for randomized clinical trials to establish causality and assess long-term effects for individuals at risk of cardiovascular diseases or cognitive decline. Incorporating salivary microbiome profiling and gastric pH measurements would also provide mechanistic insights. In addition, raising awareness among health care professionals and the general population about the potential risks associated with long-term PPI use is equally important. Increasing knowledge on this issue could lead to more informed decisions regarding PPI prescribing and use and help prevent potential adverse effects on both vascular and cognitive health. This can be achieved by incorporating qualitative research, which may help to identify knowledge gaps and guide educational strategies to promote informed PPI prescribing and use.

## 8 Conclusion

The therapeutic benefits of dietary NO₃^−^ rely on its conversion to NO in the stomach’s acidic environment. However, PPIs, which raise gastric pH, greatly reduce the efficacy of this process. This interaction may have important clinical implications, particularly for PPI users. Future research should explore how acid-suppressing drugs influence NO metabolism, especially alongside plant-based NO₃^−^ sources, and assess their impact on cardiovascular health through clinical trials.
